# Correlates of transitions from alcohol use to disorder diagnosed by DSM-5 in China

**DOI:** 10.1186/s12888-021-03413-w

**Published:** 2021-08-31

**Authors:** Tingting Zhang, Zhaorui Liu, Guohua Li, Yueqin Huang, Yanxiang Li, Hongchun Geng, Hui G. Cheng

**Affiliations:** 1 Peking University Sixth Hospital, Peking University Institute of Mental Health, NHC Key Laboratory of Mental Health (Peking University), National Clinical Research Center for Mental Disorders (Peking University Sixth Hospital), Beijing, 100083 China; 2 Chifeng Anding Hospital, No. 18 Gonggeer Road, Chifeng, 024000 Inner Mongolia China

**Keywords:** Alcohol use disorder, Prevalence, First drinking, Regular drinking, Transition

## Abstract

**Background:**

This study aimed to describe the prevalence and lifetime criteria profiles of DSM-5 alcohol use disorder (AUD) and the transitions from alcohol use to disorder in Chifeng, China.

**Methods:**

Face-to-face interviews were conducted using Composite International Diagnostic Interview-3.0 (CIDI-3.0) among 4528 respondents in Chifeng.

**Results:**

The weighted lifetime and 12-month prevalence of DSM-5 AUD were 3.03 and 1.05%, respectively. Mild lifetime AUD was the most prevalent severity level (69.53%). The two most common criteria were “failure to quit/cutdown” and “drinking more or for longer than intended.” Lifetime prevalence was 65.59% for alcohol use, and 22.97% for regular drinking. Male and domestic violence were risk factors for the transition from alcohol use to regular drinking or AUD and from regular drinking to AUD. Younger age was risk factor for the transition to AUD from alcohol use or regular drinking. Poverty (OR = 2.49) was risk factor for the transition from alcohol use to regular drinking. The earlier drinkers were more likely to develop to regular drinking (OR = 2.11).

**Conclusion:**

AUD prevalence in Chifeng was not as high as that in Western countries. The study revealed that multiple risk factors might contribute to the transition across different stages of alcohol use. Further research should explore the underlying mechanisms.

## Background

Alcohol use disorder (AUD) is one of the most prevalent mental disorders and is a significant burden of global importance. A meta-analysis showed pooled 3.8% current prevalence and 3.4% lifetime prevalence globally [1]. According to the data of Global Burden of Disease (GBD) in 2017, AUD accounted for 1.26% of the total years lived with disability (YLD) in 354 diseases and accounted for 8.73% of the disease burden attributed to mental and behavioral disorders [2]. Recent epidemiological surveys also found that AUD is prevalent in China [3]. A published meta-analysis by Cheng et al. reported that the pooled estimate for current and lifetime prevalence of AUD was 3.2 and 2.5%, respectively [4]. For the GBD data of China, AUD (the second most important disease) accounted for 11.7% of the DALY attributed to mental and behavioral disorders, following major depressive disorder [5]. A survey in China conducted in 2012 showed that the prevalence of alcohol drinking in males was 57.8% and the mean daily alcohol intake level was 32.7 g, higher than the worldwide level in 2016 (53.6%, 19.3 g) [6].

Research on AUD reported a prevalence range of 0.016–5.81% in China, varying among regions [7–10], which could be partially accounted for drinking culture. Heavy drinking commonly occurs among business associates, parties with close friends, and other social events, and attendance or participation are considered the social indication of openness and sincerity, especially in northern China and some ethnic groups [6]. Hence, the prevalence rates of AUC in these regions and people are higher than the others. The variability of prevalence exists among WHO regions. The 12-month prevalence of the population aged 15 years and older was the highest in European Region (8.2%) and in the Region of Americas (8.2%), and was the lowest in the Eastern Mediterranean Region (0.8%) [11]. Lifestyle, religious belief and culture norms plays an important role in alcohol use [12].

Chifeng is a city located in the middle-eastern of the Inner Mongolia Autonomous Region, where drinking alcohol is a regular and popular activity in daily life. Most of the people in Chifeng are ethnic Han and Mongolian, accounting for 77.3 and 19.1%, respectively. No epidemiological survey had been conducted in Chifeng before 2010, and little was known about the prevalence of mental disorders in this multi-ethnic region. In 2011, a cross-sectional study on mental disorders (including anxiety disorder, mood disorder, substance use disorder, and impulse control disorder) was conducted in Chifeng using the Composite International Diagnostic Interview-3.0 (CIDI-3.0), in order to investigate the prevalence rates of mental disorders, and social and psychological correlates, providing insights for future policies and practices. The study revealed a 2.5% lifetime prevalence of AUD by the Diagnostic and Statistical Manual of Mental Disorders, Fourth Edition (DSM-IV), following major depressive disorder and specific phobia [13]. In 2010, DSM-5 criteria of AUD were proposed. Eleven criteria, including 10 criteria of DSM-IV (dropping “legal problems”) and a new criterion (adding “craving”), are used to diagnose AUD instead of differentiating between alcohol dependence and abuse in DSM-5. Individuals who endorse sub-threshold numbers of symptoms of dependence, but none for abuse, are excluded by DSM-IV, but could be diagnosed AUD positively in DSM-5. Thus, we proposed to estimate the prevalence of AUD and clinical features based on DSM-5 criteria using the data of the survey in 2011.

Despite estimating the prevalence and clinical feature of AUD of DSM-5, we proposed to explore the correlates of transitions between the different stages of alcohol use problems. In recent years, a growing body of research on AUD has focused on first drinking, regular drinking, and the transition to AUD [14–17]. Research suggested that a person’s age at the first use of alcohol was a potentially powerful predictor of the progression to alcohol-related harm, indicating that the earlier the age at first drinking, the greater the risk of developing serious problems such as AUD [15, 18]. However, the association strength between age and AUD risk was challenged by conflicting results [19, 20]. Raul Caetano et al. found that age at first drinking was not associated with AUD among non-border Mexican Americans and South/Central Americans [16]. Currently, research on the age at first drinking is limited in China [21], and among which fewer have focused on the transition to AUD [22]. Moreover, some family members commonly make fun of children, including babies, to taste some alcohol with chopsticks in China, and some researchers considered an event like this to be their first use of alcohol [23]. Whereas, the role of this alcohol taste in transition to AUD was not examined. Even, whether this taste at an early age could be recognized as first drinking is still worth of consideration. In addition to the age at first drinking, related research showed that risk factors of transition to AUD included being male, younger cohorts, and lower education level. Childhood adversities were also well documented to be associated with drinking problems and the transition [20, 21, 24, 25]. Generally, there existed several researches on transition to AUD in China; while none was caught on in Chifeng, a city with a comparatively more entrenched drinking culture. Considering the related information of the research, including the diagnosis of AUD, the age of alcohol use, some demographic variables, and childhood adversities, could be yielded from CIDI, the epidemiological survey in Chifeng mentioned above provided an opportunity to understand the transition to AUD in the area and make comparisons with other studies.

The aims of the study were to investigate the prevalence of AUD by DSM-5 criteria and the clinical feature in Chifeng, and to explore the correlates of transitions in different stages of alcohol use problems.

## Methods

### Sample and data collection

The survey population of the study was a regionally representative sample of people aged 18 and over living in community households in Chifeng. The respondents with physical diseases, which affected communication, and mental disorder inpatients were excluded from the sampling population. A three-stage sample strategy was implemented in the survey. The first stage of sampling was to select communities or villages using proportionate to size sampling (PPS). The households were selected using systematic sampling in the second stage. The number of selected households was inflated according to the estimated response rate. In the third stage, the respondents were selected from the household using the Kish table sampling [26]. Overall, 6376 respondents from 57 communities and 51 villages were investigated [13].

All of the interviewers were trained uniformly in one week and passed the quiz. Face-to-face interviews were conducted through November 2011 to April 2013. The study was approved by the Ethical Committee of the Sixth Hospital of Peking University. All participants were provided informed consents and signed the consent forms prior to their participation in the study.

### Measures

#### Instrument

CIDI-3.0 was a fully structured interview instrument administered by trained lay interviewers. The instrument could generate diagnosis with the definitions and criteria of the DSM-IV diagnostic systems from the survey data. It has been used by the World Mental Health Surveys in over 30 countries [27]. CIDI-3.0 was translated from English into Chinese with cultural adaptation and localization. Huang et al. evaluated the test-retest reliability and validity of the Chinese version of CIDI-3.0. By clinical reappraisal, the instrument was found to have a good to excellent validity and reliability, which indicated CIDI-3.0 was acceptable as a validated instrument for survey on mental disorders [28]. CIDI-3.0 was implemented in two parts because it was quite a long interview instrument. Part I included the core diagnosis (i.e., depression, anxiety, AUD), whereas Part II interview included some additional topics (i.e., post-traumatic stress disorder, attention deficit hyperactivity disorder) and other suspected correlates. The Part I interview was conducted in all respondents. Considering the time-consumption and high comorbidity among mental disorders, the Part II interview was only administered to respondents who were positive for certain symptoms and suspected to have core diagnoses in Part I interview, which was generated by the computer-assisted personal interview system based on the answers of the Part I interview, and a 25% random sample drawn from the rest of the respondents [27]. A total of 4528 respondents completed Part I interview, and 2102 completed Part II interview.

#### Alcohol use and the age at first drinking

Respondents were asked, “How old were you the very first time you ever drank an alcoholic beverage, including beer, wine, wine coolers, and hard liquor like vodka, gin, or whiskey?” The identified age was reported. The individuals who reported never were considered as lifelong abstainers. The alcohol users were divided into two groups according to the age at first drinking as early drinkers (< 18 years) and late drinkers (≥18 years).

#### Regular drinking

The respondents who admitted to having drunk alcoholic beverages were asked: “How old were you when you first started drinking at least 12 drinks in a 12-month period?” The positive responders were identified as regular drinkers [29]. If the respondent could not recall the exact age, a question “was it before your teens” would be asked. If the respondent answered “yes”, the interviewer would record the age as 12. If the respondent answered “no”, a further question would be asked “was it before your twenties”. The age would be set as 19 when the respondent said “yes”. Otherwise, the age would be set as 20 years old. The regular drinkers were divided into two groups according to the age when they started drinking regularly as early regular drinkers (< 18 years) and late regular drinkers (≥18 years).

#### AUD

The AUD was diagnosed using CIDI-3.0 in accordance with DSM-5. The individuals who reported two or more positive indicators in a 12-month period during their lifetime were identified as AUD. Severity levels were identified based on the number of positive indicators: mild (2 or 3 indicators), moderate (4 or 5 indicators), and severe (6 or more indicators). If the respondents admitted to having two or more symptoms, they would be asked “Can you remember your exact age the very first time you had either/any of these problems? How old were you?”. The reported age was set as the age of onset of AUD. The way to deal with the respondent who forgot the exact age was similar to regular drink above.

#### Other correlates

Sociodemographic correlates included gender, age (defined by age at interview in categories 18–34 years, 35–49 years, 50–64 years, and ≥ 65 years), marital status (married/cohabitating and unmarried), and education level (none, primary school, junior high school, senior high school and more). In the CIDI-3.0, information of education years and age of marriage were considered as time-varying covariates in the analysis.

Despite AUD, other mental disorders were also diagnosed by CIDI-3.0. Mental disorders which preceded first drinking included mood disorder (depression, bipolar) or anxiety disorder (social phobia, agoraphobia, panic, specific phobia, specific phobia, obsessive-compulsive disorder, generalized anxiety disorder, and attention deficit hyperactivity disorder), which were diagnosed before the onset of alcohol use.

Childhood adversity variables were dysfunctional family (including parental alcohol use, parental mental problems, and divorce of parents), poverty, domestic violence, and neglect or lack of childhood support (including child neglect and poor relationship with parents). All variables were based on respondents’ self-reports. The respondents who refused to answer the questions or answered “don’t know” would be coded as “no”.

### Data analysis

Cumulative probability of the lifetime prevalence of alcohol use, regular use, and AUD were estimated using the two-part actuarial method, and the differences were examined using the log-rank test.

To indicate the predictors of transitions across the three stages of alcohol use, discrete-time survival analysis by the logit function with person-year was used [30]. Odds ratios (ORs) and significance values were estimated by the Taylor series linearization method. The person-year data array analyzed transition from alcohol use to regular drinking included all years from first drinking to regular drinking. For the respondents who had not changed to regular drinking, the array ended at the age of interview. For the person-year data array from regular drinking to AUD and from alcohol use to AUD, the dataset was constructed similarly. When data was constructed, logistic regression analysis was performed on the person-year data. The dependent variable (coded as 1 for present event and 0 for absence event) was regressed based on the predictors and time dummy variables. The survival analysis accounted for the possibility that respondents who had not transitioned to AUD might do so in future. The correlates included age at first drinking/regular drinking, gender, age at interview, mental problem preceding the first drinking, dysfunctional family, poverty, domestic violence, neglect or lack of childhood support, marital status (time-varying), education level (time-varying), and person-year (time-varying). Considering the norm of alcoholic taste in China mentioned above, some respondents might be regard the taste experience as first alcohol use. Given this situation, the distribution of age at first alcohol use was analyze, and 362 respondents reported using alcohol before 6 years old. In general, those people were not really identified as alcohol user at such a young age in this situation. Therefore, those people when addressing the correlates of transitions between the different stages of alcohol use problems were excluded. Sample weights were calculated in consideration of the different probabilities of selection, gender, and age distribution in Chifeng. SAS 9.4 was used for all analyses. The prevalence rates were adjusted for clustering and weighting. *P* < 0.05 was set as statistically significant.

## Results

### Demographic information

Among the 4528 participants, 46.1% were men, 93.1% were married, 32.8% lived in the urban area, and the average age was 48.1 ± 13.6 years. A total of 17.6% of the respondents had finished at least high school education. However, only 28.9% of the respondents reported being employed, while the other 71.7% were students, housewives, retired, unemployed, and involved in other activities.

### Prevalence of AUD by DSM-5

In total, 159 individuals met AUD criteria of DSM-5 over the course of their lifetime. The weighted lifetime and 12-month prevalence of AUD were 3.03 and 1.05%, respectively. The proportion of respondents with either a severe lifetime AUD (11.07%) or a moderate disorder (19.40%) was generally less than that of respondents with a mild disorder (69.53%). The median onset age of lifetime AUD was 27 years old (P_25_: 23 vs P_75_: 35).

Table 1 shows the prevalence of lifetime criteria among AUD according to severity level. Among the respondents with AUD, the two most prevalent criteria were “failure to quit/cutdown” and “drinking more or for longer than intended”. The two lowest criteria were “giving up” or “reducing activities (neglect activities)” and “spending a great deal of time to obtain or use alcohol, or recover from its effects (time spent)”. The same results were found among the mild group and the moderate and severe group. For all criteria, the proportions of moderate and severe group were statistically significant than those of mild group (*P* < 0.05).

**Table 1:** Prevalence of lifetime criteria according to the DSM-5 based on severity level (%)

Criteria	Overall AUC (*n* = 159)		Mild (*n* = 110)		Moderate and severe (*n* = 49)		*P* (mild v.s. moderate and severe)
	%	*SE*	%	*SE*	%	*SE*	
Drinking more or for longer than intended	48.37	4.01	38.25	4.97	71.47	7.66	0.002
Failure to quit/cutdown	54.68	5.21	44.83	5.52	77.88	9.03	0.005
Time spent	11.94	3.58	6.33	3.04	24.66	8.40	0.010
Craving	34.92	4.68	24.26	5.09	59.25	8.20	< 0.001
Neglect obligation	43.47	4.46	36.29	5.55	59.86	6.56	0.007
Interpersonal problems	18.49	3.29	11.94	2.83	33.44	7.72	0.001
Neglect activities	8.69	2.33	4.00	1.69	19.39	5.72	< 0.001
Hazardous use	30.82	3.40	23.90	4.44	46.62	6.51	0.008
Health activities	22.51	3.47	14.89	3.03	39.88	6.66	< 0.001
Tolerance	21.76	3.31	12.10	3.63	43.81	6.54	< 0.001
Withdrawal	23.04	3.87	13.80	3.21	44.01	7.86	< 0.001
Overall AUC	–	–	69.53	4.11	30.47	4.11	–

### First drinking, regular drinking, and transition to AUD

**Fig. 1:**
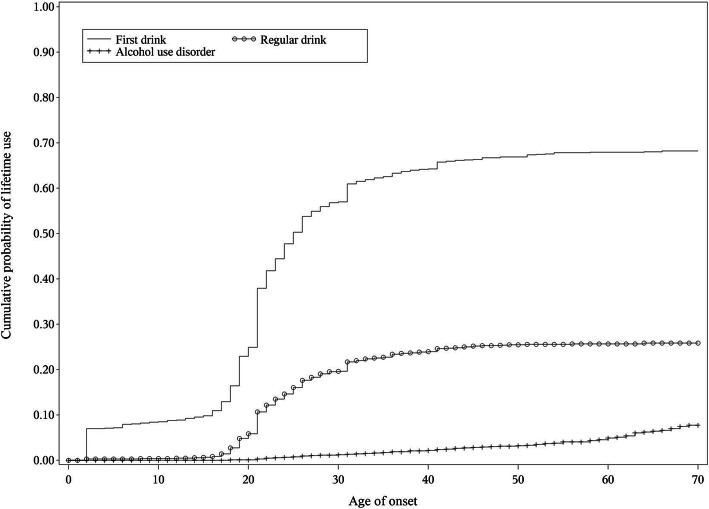
Cumulative lifetime probability of first drinking, regular drinking, and alcohol use disorder

In total, 2966 (65.59%, SE = 1.43) of the participants reported having drinking histories; only 1091 (22.97%, SE = 1.24) respondents reported regular drinking during their life. The median onset ages of first drinking and regular drinking were 20 years old (P_25_: 17 vs P_75_: 24) and 21 years old (P_25_: 20 vs P_75_: 27), respectively. Figure 1 shows the cumulative lifetime curves of first drinking, regular drinking, and AUD. Majority of the respondents reported having their first drinking and regular drinking experience at less than 30 years of age.

For the discrete-time survival analysis, the model was adjusted for several sociodemographic variables, comorbidity with other mental disorders preceding first drinking, and childhood adversity. Table 2 presents the ORs derived from discrete-time survival analyses of stage transitions from alcohol use to AUD. Generally, male, younger age and domestic violence were significantly associated with the transition stage. The transition from alcohol use to regular drinking was associated with poverty (OR = 2.49). Early drinking behavior was a risk factor of the transition from alcohol use to regular drinking (OR = 2.11).

**Table 2:** Correlates associated with transitions across alcohol use stages [OR (95%CI)]

		Transition to regular drinking from alcohol use (*n* = 2600)	Transition to AUD from alcohol use (*n* = 2600)	Transition to AUD from regular drinking (*n* = 1052)
First drinking/regular drinking	Early	2.11 (1.66–2.67)*	1.35 (0.77–2.34)	0.94 (0.54–1.64)
	Late	Ref	Ref	Ref
Gender	Female	Ref	Ref	Ref
	Male	3.13 (2.49–3.95)*	18.57 (5.80–59.48)*	9.18 (2.80–30.09)*
Age at interview	18–34	Ref	Ref	Ref
	35–49	0.78 (0.57–1.06)	0.50 (0.21–1.18)	0.42 (0.18–0.99)*
	50–64	0.86 (0.63–1.17)	0.37 (0.15–0.92)*	0.27 (0.11–0.66)*
	≥65	0.69 (0.46–1.03)	0.07 (0.02–0.26)*	0.06 (0.02–0.25)*
Marital status	Unmarried	Ref	Ref	Ref
	Married/cohabiting	0.96 (0.71–1.31)	1.51 (0.58–3.94)	1.50 (0.56–4.03)
Educational level	None	Ref	Ref	Ref
	Primary	1.16 (0.82–1.63)	1.27 (0.51–3.16)	1.19 (0.45–3.19)
	Junior high school	1.19 (0.83–1.71)	1.66 (0.65–4.22)	1.57 (0.57–4.31)
	Senior high school and more	1.30 (0.88–1.90)	1.40 (0.51–3.81)	1.39 (0.48–4.08)
Mental problem preceding the first drink	Yes	1.20 (0.90–1.61)	1.73 (0.92–3.28)	1.50 (0.79–2.86)
	No	Ref	Ref	Ref
Dysfunctional family	Yes	1.14 (0.85–1.53)	1.72 (0.96–3.07)	1.52 (0.84–2.75)
	No	Ref	Ref	Ref
Poverty	Yes	2.49 (1.05–5.91)*	1.01 (0.13–7.92)	0.68 (0.09–5.33)
	No	Ref	Ref	Ref
Domestic Violence	Yes	1.71 (1.14–2.58)*	3.05 (1.50–6.20)*	2.24 (1.08–4.64)*
	No	Ref	Ref	Ref
Neglect or lack of childhood support	Yes	0.77 (0.52–1.13)	0.62 (0.28–1.37)	0.84 (0.37–1.88)
	No	Ref	Ref	Ref

*AUD* Alcohol use disorder, *OR* Odds ratio, *CI* Confidence interval;

*Significant OR (*P* < 0.05)

## Discussion

In the current study, the prevalence of AUD in Chifeng diagnosed by DSM-5 (replacing the previous DSM-IV) was first described, and few reports were reported nowadays in China. It is a meaningful attempt because there were some data of AUD diagnosed by DSM-IV in the past. Meanwhile, the age at first drinking, regular drinking, and the transition to AUD were investigated, exploring the influence of age of alcohol use, regular drinking, and other variables on the transition to AUD.

In the study, the lifetime AUD prevalence of 3.03% and 12-month prevalence of 1.05% were noted; these rates were lower than those reported in some similar prevalence surveys from other areas in China [8, 9, 31] and other countries [32, 33]. The data of WMHS in Beijing and Shanghai revealed the lifetime and 12-month prevalence rates of 4.9 and 1.6%, respectively [8]. Another epidemiological survey in Liaoning province, China, which is next to Inner Mongolia, reported the lifetime prevalence of 3.94% [34]. The prevalence rates of lifetime alcohol use and regular drinking were 65.59 and 22.97%, respectively. The rate of alcohol use resembled that from a report of another survey of two metropolitan Chinese cities [22], USA [35], and Singapore [36]. However, the prevalence of regular drinking was much lower than those reported in USA (72.9%) [29], Brazil (56.2%) [37], and Singapore (32%) [36]. There are several possible reasons for the lower prevalence. One reason could be concealment during self-report. Some alcohol users might deny alcohol abuse or understate real issues. Several studies concluded that self-reported data on substance use, human immunodeficiency virus risk behaviors, especially during face-to-face interviews, had a number of challenges in terms of validity and reliability [38–40]. Self-reported alcohol use often lacked agreement with the results of alcohol use estimated using biomarkers, particularly in women [41, 42]. This could be partially avoided by other kinds of instruments, like the Structured Clinical Interview for DSM–IV (SCID), which is conducted by trained psychiatrists with the presence of a family member. The self-reported drinking could also be influenced by local customs. The accuracy of alcohol use reporting always varied in population, particularly in a group of people who often drank [42]. With a 65.59% prevalence of alcohol use, drinking is common in Chifeng. Therefore, we speculated that AUD would be underestimated.

The two most prevalent criteria among different severities were “failure to quit/cutdown” and “drinking more or for longer than intended”. This replicates the findings of many related survey analyses [43–45]. In contrast to “failure to quit/cutdown” and “drinking more or for longer than intended” criteria, neglect activity and spent time were the two criteria with the lowest prevalence across severities. Preuss et al. used item response theory model to estimate the severity of an AUD criterion, with high severity being that criterion endorsed less frequently by respondents. The research showed that the highest severity scores were found in the spent time and neglect activity, which is consistent with our findings [46]. Differential item functioning analyses of the research from four countries showed that the time spent drinking was considered as a severity and discrimination indicator [47].

In the present study, gender was a consistent predictor of transition across different stage of alcohol use. The results of the ORs of different stages of alcohol use showed that more males transited to AUD than to regular drinking from alcohol use. According to the report from World Health Organization, gender ratio (male/female ratio) in heavy episodic drinking among drinkers were higher than that for the prevalence of current drinking [11]. It meant that not only fewer women drank than men, but when they drank, they drank less and exhibited heavy episodic drinking less often. Younger people had higher risk of transitioning into AUD from alcohol use and regular drinking, similar to the results of other studies in the USA [29], Singapore [36], Brazil [37], and China [22]. Contradictory results could be found in the analyses of some previous studies in China, which indicated that older age was a risk factor [48, 49]. Comparing with older people, young people were disproportionately affected by alcohol. With regard to the consumption, heavy episodic drinking peaked in the age of 20–24 in all regions of the world [11]. The results of China Mental Health Survey showed that the people in the age of 18–34 suffered the highest prevalence of AUD, compared with people in other age groups [3]. In patients with severe mental disorders, the prevalence of AUD was higher among younger adults (18–25 years) than older ones [50]. Substantial evidence showed that adolescents with a history of alcohol use differ neurally and cognitively from other adolescents. Alcohol use in adolescents often affected attention, verbal learning, visuospatial processing and memory and altered development of grey and white matter volumes [51]. The current study also showed that domestic violence was associated with the transition across the stages of alcohol use, and poverty was associated with the transition from alcohol use to regular drinking. In a previous research, childhood adversities, including divorce of parent, chronic tension in the household, inter-parent violence, child neglect, and harsh physical punishment, had been associated with the occurrence of AUD [21, 24, 25]. The present study showed that early drinkers were more likely to develop to regular drinking than late drinkers. The findings are similar with previous literature indicating that respondents with the earlier age of drinking were more likely to develop to alcohol use problems [52, 53]. However, the age at first alcohol drinking and regular drinking was not associated with the transition to AUD, which was in agreement with another analysis from a sample in China [22]. An US study found that early age of first alcohol use and regular drinking were risk factors for the transition to alcohol abuse and dependence [29]. Some previous studies in western countries also found a positive association between early age at first drinking and drinking severity [52, 54]. The negative results in the Chinese population might be explained by the special culture drinking customs in China. The norms in most Chinese people encourage social and celebratory drinking but discourage daily solitary drinking [55, 56], thereby partially reducing risk of AUD, especially severe use. The negative association could also be explained at a genetic level. Luczak et al. found that in Chinese individuals, age at first drinking as a risk factor for AUD was moderated by an alcohol-metabolizing gene ALDH2*2 [57].

Several limitations of the research should be considered. First, data were collected in Chifeng so the findings cannot be generalized in other Chinese regions. Second, the sample excluded the migrant population and those who were institutionalized. However, previous studies showed that certain populations might exhibit a higher prevalence of alcohol use than the general population [49, 58]. Third, recall bias could exist because the ages of onset in different stages of alcohol use were obtained through retrospective estimates [22, 29, 36]. Special efforts were made in the CIDI-3.0 to help individuals recall the age of onset [27]. One way was to decompose the questions to mimic the memory search strategies successfully. Despite the dating error, respondents tended to report the age of onset as being more recent than it actually was [59]. In the discrete-time survival analysis, the age of early drinking was set as a categorical variable to control bias, although recall bias could lead to some misclassification in this way. For the respondents who could not recall the exact age of onset, a series of probes were set to help them as mentioned above. For instance, one probe was “was it before your twenties?” If the respondents answered “yes,” 20 was used in the analysis; if the respondents answered “no,” another probe would be asked. Fourth, the current study only focused on a limited number of sociodemographic variables and childhood adversities. A previous study showed considerable variation in age at first drinking and drinking across national groups [16, 60, 61]. Therefore, the effect of age on first drinking might vary in different ethnicities in China. However, information of people from different ethnic backgrounds was regrettably not collected. Fifth, as this survey is a cross-sectional study, it cannot provide definite evidence of a causal link between the age of alcohol use and related problems. However, a population-based twin study indicated that the association between early age of alcohol use and AUD in later life did not reflect a causal relationship, but was rather due to common genetic risk factors [62]. Therefore, longitudinal investigations are needed to confirm the current findings and explore the underlying mechanisms.

## Conclusion

The study provided the prevalence of AUD by DSM-5 and lifetime criteria based on the severity level in Chifeng, China. The research also demonstrated the correlates, especially gender, age at interview, and the age at first drink and age at regular drinking, which contribute to the transition among different stages of alcohol use. The global strategy to reduce harmful use of alcohol represents international consensus. The age of alcohol use deserves serious attentions, as well as the alcohol consumption. The study raises important policy implications for the development of regular drinking and AUD prevention for youth. It was demonstrated that the prevention focused on youth would avert substantial alcohol related harm. More attentions should still be paid to the male. Policy makers should pay more attention to the individuals from families in poverty or with violence.

## Data Availability

The data that support the findings of this study are available from the corresponding author on request.

## Abbreviations

AUD: Alcohol use disorder
GBD: Global Burden of Disease
YLD: Years lived with disability
CIDI-3.0: The Composite International Diagnostic Interview-3.0
DSM-IV: The Diagnostic and Statistical Manual of Mental Disorders, Fourth Edition
